# Mapping the Evolution of Thyroid Ultrasound Research: A 30-year Bibliometric Analysis

**DOI:** 10.2174/0115734056396607250811115439

**Published:** 2025-08-21

**Authors:** Ting Jiang, Chuansheng Yang, Lv Wu, Xiaofen Li, Jun Zhang

**Affiliations:** 1 Department of Ultrasound, Jiangxi Provincial People's Hospital, The First Affiliated Hospital of Nanchang Medical College, Nanchang 330006, China; 2 Department of Nuclear Medicine, Ganzhou Cancer Hospital, Ganzhou 341000, China; 3 Department of Radiology, Jiangxi Provincial People's Hospital, The First Affiliated Hospital of Nanchang Medical College, Nanchang 330006, China; 4 Department of Ultrasound, The Affiliated Hospital of Jiangxi University of Chinese Medicine, Nanchang, 330006, China

**Keywords:** Thyroid ultrasound, Bibliometrics, TI-RADS, Radiofrequency ablation, Deep learning, Thyroid volume

## Abstract

**Introduction:**

Thyroid ultrasound has emerged as a critical diagnostic modality, attracting substantial research attention. This bibliometric analysis systematically maps the 30-year evolution of thyroid ultrasound research to identify developmental trends, research hotspots, and emerging frontiers.

**Methods:**

English-language articles and reviews (1994-2023) from Web of Science Core Collection were extracted. Bibliometric analysis was performed using VOSviewer and CiteSpace to examine collaborative networks among countries/institutions/authors, reference timeline visualization, and keyword burst detection.

**Results:**

A total of 8,489 documents were included for further analysis. An overall upward trend in research publications was found. China, the United States, and Italy were the productive countries, while the United States, Italy, and South Korea had the greatest influence. The journal Thyroid obtained the highest IF. The keywords with the greatest strength were “disorders”, “thyroid volume”, and “association guidelines”. The timeline view of reference demonstrated that deep learning, ultrasound-based risk stratification systems, and radiofrequency ablation were the latest reference clusters.

**Discussion:**

Three dominant themes emerged: the ultrasound characteristics of thyroid disorders, the application of new techniques, and the assessment of the risk of malignancy of thyroid nodules. Applications of deep learning and the development and improvement of correlation guides such as TI-RADS are the present focus of research.

**Conclusion:**

The specific application efficacy and improvement of TI-RADS and the optimization of deep learning algorithms and their clinical applicability will be the focus of subsequent research.

## 1 INTRODUCTION

The thyroid gland, a butterfly-shaped organ located in the neck, consists of two lobes connected by an isthmus. It plays a critical role in regulating the body's metabolism through the secretion of thyroid hormones [1]. Thyroid hormones influence various physiological processes, including heart rate, body weight, and energy levels [2, 3]. Common thyroid diseases include nodular goiter, thyroiditis, thyroid adenoma, and thyroid cancer. Thyroid nodules, a manifestation of many thyroid disorders, have been detected in up to 68% of the healthy population [4]. The above thyroid-related disorders can present with clinical symptoms such as fatigue, lethargy, weight loss, fever, palpitations due to hormone disruption, or compression of structures such as the trachea due to an enlarged thyroid gland.

Ultrasound has undergone significant advancements since its inception, evolving from simple imaging techniques to sophisticated, high-resolution modalities. Its noninvasive nature, absence of ionizing radiation, and real-time imaging capabilities make it an indispensable tool in thyroid disease evaluation [5, 6]. Many scholars have explored the ultrasound manifestations of thyroiditis, thyroid cancer, goiter, benign nodules, and other diseases, making noninvasive diagnosis and follow-up possible [7, 8]. Technological innovations such as elastography and contrast-enhanced ultrasound (CEUS) have further enhanced its diagnostic accuracy, allowing for better characterization of thyroid nodules and detection of malignancies [9, 10].

Despite the numerous reviews on the clinical application of ultrasound in the thyroid, there is a noticeable gap in the literature concerning the quantitative analysis of research trends and the impact of ultrasound technology over time, which can be effectively addressed through bibliometric studies [11]. Understanding these trends will provide valuable insights into the trajectory of ultrasound technology and its clinical applications, guiding future research directions. The primary objective of this study is to conduct a comprehensive bibliometric analysis to trace the evolution of research in this field, filling the existing gap in the literature and contributing to a more nuanced understanding of its role in thyroid evaluation.

## 2 MATERIALS AND METHODS

### 2.1 Literature Search

Studies on the application of ultrasound in thyroid tissue published between 1994 and 2023 were identified in the Web of Science Core Collection (WoSCC) database [12]. The search strategy was as follows: (TS = (Ultrasound AND thyroid)) AND FPY = (1994--2023). This search yielded 8,806 relevant documents. The search results were then filtered to include only articles and review articles written in English [12]. To ensure the relevance of the data, studies that did not pertain to a specific research area were excluded.

The remaining documents were exported in “txt” format for further analysis. Additionally, the Web of Science automatically generated data on the number of publications and H-index for each country, institution, author, and journal. The H-index is a metric that combines an author’s number of publications and citations, where a higher H-index indicates greater impact. The data search and document download were completed on May 22, 2024.

### 2.2 Data Analysis

Bibliometric analyses were performed *via* CiteSpace (version 6.3.1) and VOSviewer (version 1.6.20). Microsoft Excel 2024 was used to create a trend graph of annual publication volumes. VOSviewer was employed to analyze collaboration among countries/regions, institutions, and authors. CiteSpace was used to identify research hotspots through burst keyword analysis and reference timeline view.

Burst keyword analysis associates keywords with high frequencies during specific periods, marking the appearance and bursts of each keyword on a line. Different colors on the line denote various meanings: light blue indicates the period before a keyword appears, red denotes the burst period, and blue signifies the period when the keyword appears without a burst.

The reference timeline view is constructed on the basis of the reference cluster. This analysis clusters the references, generates a name for each cluster automatically, and then displays the most frequently cited references within each cluster. Each root line on the timeline view represents a cluster, with the cluster name on the far left. Round nodes on the line represent references within the cluster, and lines between nodes indicate the co-occurrence of references.

## 3 RESULTS

### 3.1 Publication Volume Analysis

The initial search yielded 9,719 documents in the WoSCC. After 20 retracted documents were excluded, 769 documents were excluded because of document type, 343 were excluded because of language, and 98 were excluded because of unrelated research areas. A total of 8,489 articles and review articles written in English were included for further analysis. The flowchart of literature selection is shown in Fig. (**1**). Fig. (**2**) shows the annual publication volume and cumulative publication volume, indicating an overall upward trend in research publications in the field of thyroid ultrasound evaluation.

### 3.2 Analysis of Countries, Institutions, and Authors

Data on countries, institutions, and authors were obtained from the WoS website. Table **1** summarizes the top 10 countries/regions and institutions by publication volume, and Fig. (**3A** and **B**) present the collaboration network between the countries and institutions. The top three countries in terms of publication volume are China, the United States, and Italy, while the top three H-index countries are the United States, Italy, and South Korea. The top three institutions by publication volume are Yonsei University, Yonsei University Health System, and University of Ulsan, all from South Korea. Harvard University has the highest H-index. Notably, eight of the top ten authors by publication volume are also from South Korea, highlighting the country's significant impact in this field.

### 3.3 Journal Analysis

Analyzing journals can highlight the sources of research in a particular field, guiding readers on where to find relevant literature and providing important information for journal selection for manuscript submission. Table **2** shows the top 10 journals by publication volume in the field of thyroid ultrasound. Notably, the journal Thyroid obtained the highest IF and published the most papers among them.

### 3.4 Research Hotspot Analysis

Fig. (**4**) illustrates the top 25 keywords with the strongest citation bursts. This visualization shows the periods when key terms appeared or surged in frequency, which helps to identify the focus in each period. The keywords with the greatest strength were “disorders”, “thyroid volume”, and “association guidelines”. From 1994–2007, the keywords “disorders”, “gland”, “ultrasound”, “hypothyroidism”, “endemic goiter”, “graves' disease”, “children”, “goiter”, “thyroid volume”, “biopsy”, “urinary iodine”, “iodine deficiency”, and “localization” were frequently used. From 2008--2016, keywords such as “ultrasound criteria”, “criteria”, “differentiation”, “elastography”, “predicting malignancy”, “strain ratio”, and “real-time elastography” were used to search for bursts. After 2017, keywords with bursts included “risk stratification”, “association guidelines”, “Thyroid Imaging Reporting and Data System (TI-RADS)”, “guidelines”, and “deep learning”.

Fig. (**5**) shows the reference timeline distribution. Nine clusters were identified: #0 data system, #1 solitary thyroid nodule, #2 ultrasound elastography, #3 thyroid volume, #4 cancer risk, #5 ultrasound-based risk stratification system, #6 radiofrequency ablation, #7 deep learning, #8 benign thyroid nodule, and #9 thyroid nodule. Thyroid volume was the earliest cluster, followed by solitary thyroid nodules and ultrasound elastography. Deep learning, ultrasound-based risk stratification systems, and radiofrequency ablation were the latest clusters.

## 4 DISCUSSION

### 4.1 General Information

This study employs bibliometric methods to analyze 8,489 publications on thyroid ultrasound over the past 30 years, revealing an overall upward trend in publication volume. Significant contributions come from South Korea, the United States, Italy, and China. Notably, South Korea stands out, with Yonsei University leading in both publication volume and the H-index, demonstrating remarkable performance. South Korea even boasts eight out of the top ten authors by publication volume, underscoring the depth of their research. The journals Thyroid, Frontiers in Endocrinology, and Journal of Clinical Endocrinology & Metabolism published the most related literature, making them valuable resources for researchers interested in this field.

Research hotspots refer to topics that have gained much attention in a specific field. The analysis of hotspots is an important part of bibliometric research. Our studies analyzed keywords and references to obtain relevant information. The research period of the last 3 decades can be roughly divided into 3 phases with different tasks, which sequentially evolved from (1) exploring the ultrasound findings of thyroid disease to (2) identifying malignant thyroid nodules *via* novel technology, and finally to (3) developing standardized guidelines (TI-RADSs) and using deep learning.

#### 4.1.1 Phase 1: 1994--2007

This period focused primarily on exploring the ultrasound manifestations of thyroid disorders, particularly goiter. Goiter, one of the most prevalent diseases before 2000, is most commonly caused by iodine deficiency, which often has a regional distribution [13]. Iodine is a crucial component of thyroid hormone synthesis, and its deficiency can lead to thyroid enlargement and even hypothyroidism. This condition may result in delayed physical development, impaired brain growth, and weight fluctuations. Depending on whether thyroid dysfunction is present, goiters can be classified as either simple goiters or toxic goiters (Graves' disease) associated with hyperthyroidism. Thyroid enlargement is the most easily detectable manifestation of goiter and can be assessed through palpation and ultrasound. While palpation is typically used for a rough assessment, ultrasound is treated as the gold standard for thyroid volume measurement. Numerous studies have evaluated the reliability and efficacy of ultrasound in assessing goiter. Lyshchik A and colleagues demonstrated that 3D ultrasound offers high accuracy and reproducibility in thyroid volume measurement [14]. Other studies have investigated the influence of factors such as age, height, and weight on ultrasound-measured thyroid volume and have concluded that age-based evaluation is more reliable for determining significant thyroid enlargement [15]. Graves' disease in pregnant women can significantly impact newborns. Some studies have utilized fetal thyroid ultrasound evaluations and reported that certain fetuses develop goiter as early as 32 weeks. After maternal medication was adjusted, the majority of these fetuses were successfully treated [16].

#### 4.1.2 Phase 2: 2008--2016

Research has shifted toward the use of ultrasound, particularly novel technologies such as elastography, to assess the malignancy risk of thyroid nodules and determine the need for further fine-needle aspiration biopsy (FNAB) in recent years. With the widespread use of ultrasound and routine physical examinations, an increasing number of thyroid lesions, especially thyroid nodules and inflammatory diseases, have been detected [4]. Accurate assessment of the malignancy of thyroid lesions is crucial for effective disease management. Conventional B-mode ultrasound can be used to evaluate malignancy by analyzing the size, margins, and echogenicity of lesions, whereas Doppler ultrasound provides further insight into the vascular supply of lesions. Previous studies have identified irregular margins, microcalcifications, and hypoechogenicity as indicators of malignant nodules [17]. Another study suggested that tumor protrusion with intensive blood signals extending from the main tumor, as assessed by Doppler ultrasound, is a sign of malignancy [18].

Researchers have actively explored the efficacy of various novel ultrasound technologies in evaluating thyroid disorders. The development of elastography has enabled the measurement of tissue stiffness. Kim *et al*. [19] reported that the mean, maximum, and minimum values of strain elastography in papillary thyroid carcinoma (PTC) were significantly greater than those in benign nodules. Combining these findings with traditional B-mode ultrasound characteristics significantly improved the specificity of predicting PTC. CEUS, a new technology that enhances echoes by injecting microbubble contrast agents intravenously, has become a key tool in ultrasound diagnostics. One study used CEUS in conjunction with Doppler ultrasound to evaluate thyroid nodules and reported that complete washout in the late phase had a specificity of up to 92% in distinguishing between benign and malignant lesions [20]. Additionally, ultrasound not only aids in diagnosing thyroid lesions but also in assessing lymph node metastasis caused by thyroid cancer [21].

For clinically suspicious thyroid nodules, especially those identified by ultrasound, FNAB is typically required to confirm the diagnosis [22]. Ultrasound is often used to guide FNAB, improving the accuracy and efficiency of the procedure [23, 24]. Although it is the gold standard for determining the nature of thyroid lesions, it still has a certain false-negative rate and a 10% diagnostic miss rate [25]. To standardize the application of fine-needle aspiration biopsy (FNAB) in the evaluation of thyroid lesions, the Bethesda System for Reporting Thyroid Cytopathology was established. This system classifies cytological results into six categories (I-VI) based on malignancy risk stratification, thereby significantly improving diagnostic accuracy [26]. For indeterminate or high-risk lesions, repeat biopsy or surgical intervention may be required. Recent studies have suggested that a subset of category II lesions may harbor malignant potential [27], while the malignancy risk associated with category III lesions might be higher than previously recognized [28]. These findings underscore the critical need for developing more precise lesion detection and guidance techniques.

#### 4.1.3 Phase 3: 2017--2023

This phase is characterized by three main research focuses: TI-RADS, radiofrequency ablation, and deep learning. TI-RADS was established to provide radiologists with a unified, straightforward classification system to identify malignancies and guide follow-up, FNAB, or surgery. On the basis of decades of research, various societies have established TI-RADS to standardize image analysis in the evaluation of thyroid diseases. In 2009, Horvath *et al*. first proposed a standardized reporting system for thyroid ultrasound findings [29]. In 2017, the ACR TI-RADS Committee released an official version of TI-RADS, which scores nodules on the basis of five ultrasound features: composition, echogenicity, shape, margin, and echogenic foci, categorizing the malignancy risk into five levels [30]. The European Thyroid Association and Task Force Committee of the Korean Society of Thyroid Radiology also developed their own TI-RADS [31, 32].

The above guidelines provide a reference point for the ultrasound evaluation of thyroid nodules, and they differ somewhat in nuance. Therefore, many studies after 2017 compared the efficacy of TI-RADS in practical applications [33, 34]. Given that guidelines on TI-RADS are overwhelmingly based on adult thyroid manifestations, many scholars have begun to explore the efficacy of the guidelines in children and adolescents [35, 36]. Moreover, the guidelines rate the degree of malignancy risk on the basis of traditional ultrasound features of the nodule. Several studies have combined the features of thyroid nodules on CEUS and superb microvascular imaging with the TI-RADS classification and have shown that these techniques can optimize the clinical value of the TI-RADS alone [37-39].

Radiofrequency ablation has been another key focus of recent studies. First reported by Kim *et al*. in 2006 as an innovative treatment for thyroid nodules [40], radiofrequency ablation has gained attention as an alternative to open surgery, which may lead to complications such as hypothyroidism and significant trauma. Radiofrequency ablation is now widely used for treating benign solid and some cystic nodules, as well as recurrent thyroid cancer [41]. Compared with microwave ablation and laser ablation, radiofrequency ablation has the advantage of fewer complications [42]. Research has shown that it offers shorter operation times, lower costs, and avoids postoperative hypothyroidism than does surgical resection [43]. While the local recurrence and complication rates are comparable to those of microwave and laser ablation, radiofrequency ablation has shown a higher volume reduction rate than microwave ablation [44]. Notably, as another branch of thermal ablation technology, the increasingly mature High-Intensity Focused Ultrasound (HIFU) technique has gained progressive clinical adoption in recent years [45]. HIFU represents a non-invasive therapeutic modality that requires no device penetration into the human body, thereby constituting a truly incision-free treatment approach [46]. This technology was initially applied in thyroid-related applications around 2017, primarily for managing recurrent or persistent Graves' disease. In recent years, its therapeutic scope has gradually expanded to encompass ablation therapy for benign thyroid nodules, demonstrating remarkable efficacy coupled with favorable safety profiles. Furthermore, the HIFU-derived tissue fragmentation technique, termed histotripsy, employs short-pulse ultrasonic waves to generate microbubbles that mechanically disintegrate target tissues to subcellular levels while preserving adjacent structures. The safety of this modality has been validated in live animal models, suggesting promising potential for future clinical translation in human therapeutics [47].

Under the premise of a high thyroid lesion detection rate, radiologists face heavy workloads. Radiologists’ variability in experience and interpretation can lead to differences in assessment results, potentially resulting in unnecessary FNABs. Deep learning has revolutionized medical imaging by enabling automated analysis and interpretation of complex datasets. It leverages the increasing computational power of GPUs to identify abstract and intricate imaging features. Deep learning has been applied to thyroid ultrasound for nodule recognition and the diagnosis of various lesions. Sun *et al*. developed a deep learning network that automatically segmented thyroid nodules [48]. Zhang Q *et al*. trained a deep learning model on ultrasound images from 17,934 patients to automatically identify Hashimoto's thyroiditis, with diagnostic accuracy surpassing that of radiologists [49]. Another study involved 426 patients to evaluate the performance of an artificial intelligence system in diagnosing thyroid nodules and reported that it could be a potential decision-making tool for effectively improving the diagnostic efficiency of junior radiologists [50]. Additionally, deep learning can learn from ultrasound report texts to extract information and identify potential thyroid cancers from unstructured reports [51].

### 4.2 Limitations

Several limitations must be acknowledged in this study. Firstly, the study relies on an article and a review article written in English from the WoSCC; however, while it is comprehensive, it may not capture all relevant publications. Articles published in non-indexed journals or those in languages other than English might be underrepresented. Secondly, the analysis of keyword bursts and trends is inherently dependent on the accuracy and consistency of keyword assignment in publications. Variations in terminology and differences in keyword indexing practices across journals might affect the results. Thirdly, the division of the study period into three distinct phases is somewhat arbitrary and may not fully capture the nuances of research progression. Significant developments could span these periods or not fit neatly within them. Fourthly, while the study highlights contributions from specific countries/regions and institutions, it might overlook the efforts and advancements from smaller or less prolific research centers.

## CONCLUSION

Our bibliometric analysis suggests that thyroid ultrasound remains a hot research topic. South Korea and the United States are the most influential countries in this field. In general, the purpose of research in this field in the past 30 years has gradually shifted from ultrasonic characteristic analysis of thyroid lesions, especially goiters, to malignant risk assessment of thyroid nodules, during which the application of new ultrasound technologies such as elastography and CEUS has developed. To evaluate the risk of malignancy of thyroid nodules, many versions of TI-RADS have been proposed in recent years. Deep learning, as an objective tool for image and data analysis, has demonstrated great potential in assisting thyroid lesion identification and diagnosis. It is believed that in the future, the specific application efficacy and improvement of TI-RADS and the optimization of deep learning algorithms and their clinical applicability will be the focus of subsequent research.

## Figures and Tables

**Fig. (1):**
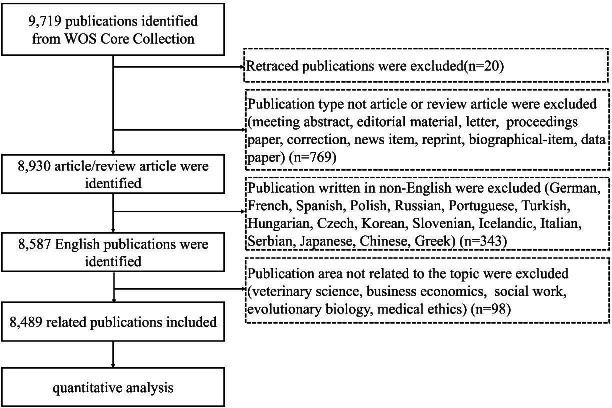
Flowchart of literature screening and article summary.

**Fig. (2):**
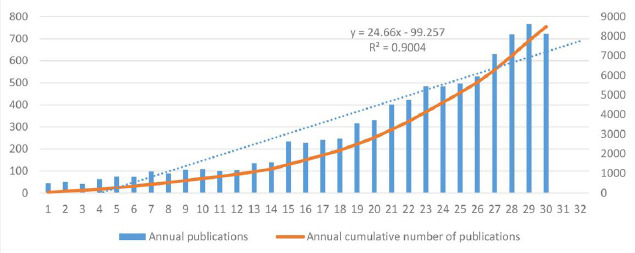
Annual and cumulative number of publications.

**Fig. (3):**
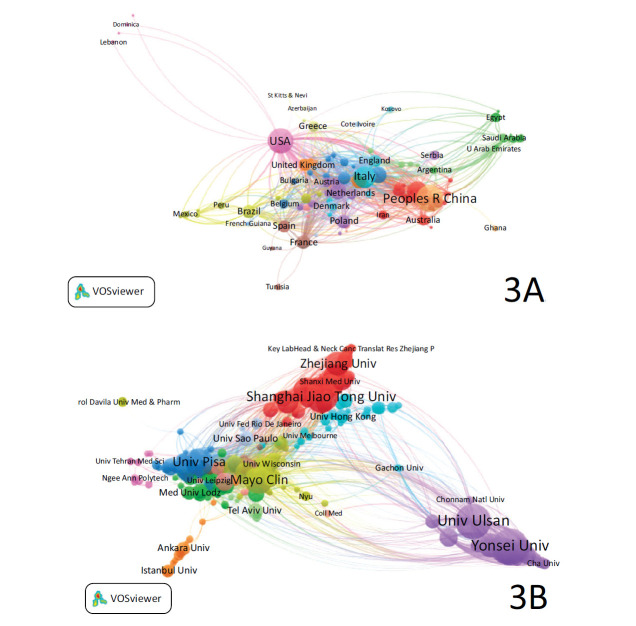
Mapping of cooperation between (**A**) countries/regions and (**B**) institutions. *The size of the nodes in the network indicates the publication volume, and the thickness of the lines between nodes represents the frequency of collaboration.

**Fig. (4):**
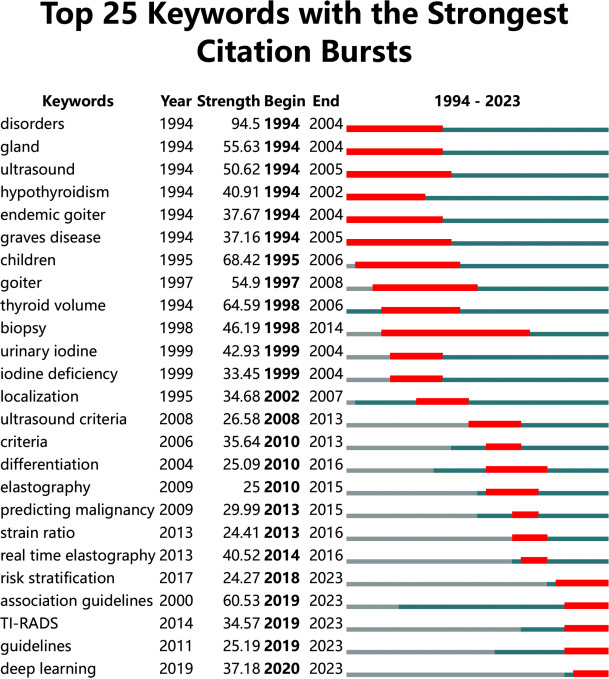
The top 25 keywords with the strongest citation bursts.

**Fig. (5):**
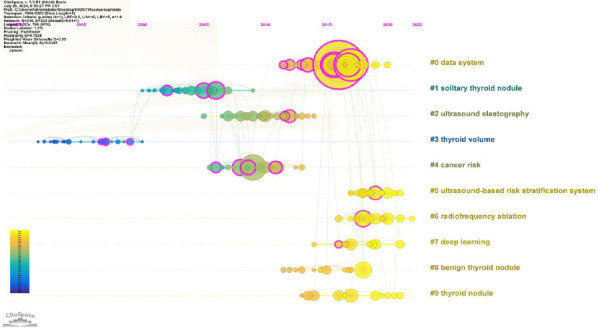
Timeline view of reference.

**Table 1:** Top 10 productive countries/regions, institutions, and authors.

Rank	Country/ Region	Count	H-index	Institution	Count	H-index	Author	Country/ Region	Count	H-index
1	China	2041	65	Yonsei University	202	46	Kwak, JY	South Korea	145	47
2	United States	1617	110	Yonsei University Health System	197	46	Baek, JH	South Korea	145	55
3	Italy	977	90	University of Ulsan	191	49	Kim, EK	South Korea	142	61
4	South Korea	776	77	Harvard University	173	53	Moon, HJ	South Korea	104	43
5	Germany	439	62	Shanghai Jiao Tong University	170	27	Yoon, JH	South Korea	79	37
6	Turkey	403	36	Sapienza University Rome	164	46	Trimboli, P	Switzerland	76	41
7	Japan	297	49	University of Pisa	140	43	Lee, JH	South Korea	73	52
8	England	249	44	Mayo Clinic	137	43	Kim, DW	South Korea	66	29
9	France	241	54	Asan Medical Center	122	43	Xu, H	China	64	52
10	Poland	213	28	University of California system	121	37	Shin, JH	South Korea	56	40

**Table 2:** Top 10 journals in terms of the number of publications.

**Rank**	**Journal**	**Publications**	**JCR**	**IF(2023)**
1	Thyroid	414	Q1	5.8000
2	Frontiers in Endocrinology	208	Q2	3.9003
3	Journal of Clinical Endocrinology & Metabolism	198	Q1	5.0002
4	Journal of Ultrasound in Medicine	189	Q2	2.1001
5	Endocrine	163	Q1	2.9997
6	Ultrasound in Medicine and Biology	158	Q2	2.4002
7	Clinical Endocrinology	151	Q2	2.9997
8	Journal of Endocrinological investigation	124	Q2	3.9003
9	European Journal of Endocrinology	121	Q1	5.2999
10	Medicine	119	Q2	1.3000

## Data Availability

The data that support the findings of this study are available from the corresponding authors [X.L.] and [J.Z.] upon reasonable request.

## LIST OF ABBREVIATIONS

WoSCC: Web of Science Core Collection
TI-RADS: Thyroid Imaging Reporting and Data System
CEUS: Contrast-enhanced ultrasound
FNAB: Fine-needle aspiration biopsy
PTC: Papillary thyroid carcinoma
HIFU: High-Intensity Focused Ultrasound
